# How the Innate Immune DNA Sensing cGAS–STING Pathway Is Involved in Autophagy

**DOI:** 10.3390/ijms222413232

**Published:** 2021-12-08

**Authors:** Wanglong Zheng, Nengwen Xia, Jiajia Zhang, Nanhua Chen, François Meurens, Zongping Liu, Jianzhong Zhu

**Affiliations:** 1 Comparative Medicine Research Institute, Yangzhou University, Yangzhou 225009, China; wanglongzheng@yzu.edu.cn (W.Z.); 13089071533@163.com (N.X.); zjj1608291303@163.com (J.Z.); hnchen@yzu.edu.cn (N.C.); 2 College of Veterinary Medicine, Yangzhou University, Yangzhou 225009, China; 3 Joint International Research Laboratory of Agriculture and Agri-Product Safety, Yangzhou 225009, China; 4 Jiangsu Co-Innovation Center for Prevention and Control of Important Animal Infectious Diseases and Zoonoses, Yangzhou 225009, China; 5 BIOEPAR, INRAE, Oniris, 44307 Nantes, France; francois.meurens@inra.fr; 6 Department of Veterinary Microbiology and Immunology, Western College of Veterinary Medicine, University of Saskatchewan, Saskatoon, SK S7N 5E2, Canada

**Keywords:** cGAS, STING, autophagy, innate immunity, IFN, DNA sensing

## Abstract

The cGAS–STING pathway is a key component of the innate immune system and exerts crucial roles in the detection of cytosolic DNA and invading pathogens. Accumulating evidence suggests that the intrinsic cGAS–STING pathway not only facilitates the production of type I interferons (IFN-I) and inflammatory responses but also triggers autophagy. Autophagy is a homeostatic process that exerts multiple effects on innate immunity. However, systematic evidence linking the cGAS–STING pathway and autophagy is still lacking. Therefore, one goal of this review is to summarize the known mechanisms of autophagy induced by the cGAS–STING pathway and their consequences. The cGAS–STING pathway can trigger canonical autophagy through liquid-phase separation of the cGAS–DNA complex, interaction of cGAS and Beclin-1, and STING-triggered ER stress–mTOR signaling. Furthermore, both cGAS and STING can induce non-canonical autophagy via LC3-interacting regions and binding with LC3. Subsequently, autophagy induced by the cGAS–STING pathway plays crucial roles in balancing innate immune responses, maintaining intracellular environmental homeostasis, alleviating liver injury, and limiting tumor growth and transformation.

## 1. Introduction

**Figure 1:**
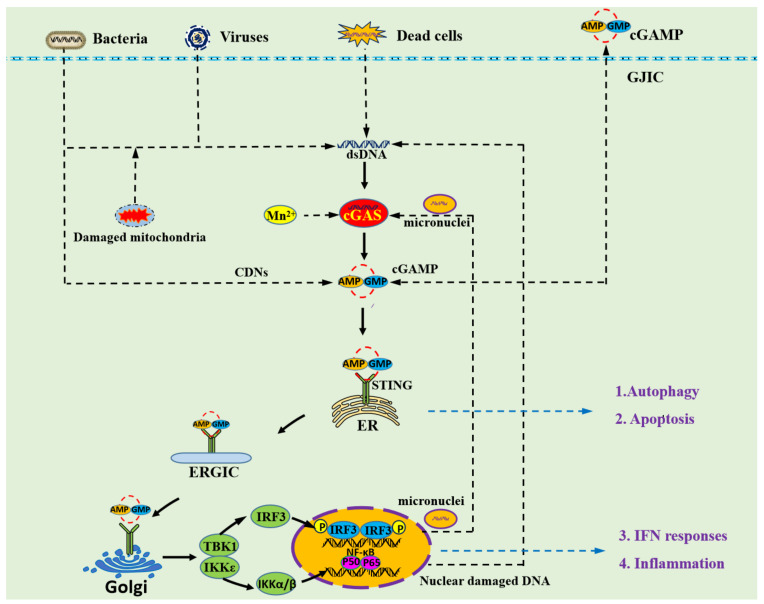
Overview of the cGAS–STING signaling pathway. The cytosolic DNA receptor cGAS could be activated by both non-self DNA and self DNA including microbial DNA, released mitochondrial DNA, cytosolic micronuclei, and aberrant chromosomal DNA. Mn^2+^ is also able to activate cGAS, leading to an unconventional catalytic synthesis of 2′3′-cGAMP [19]. The second messenger 2′3′-cGAMP and other cyclic dinucleotides (CDNs) could cross the cell membrane through gap junction intercellular communication (GJIC) [20]. Then, 2′3′-cGAMP binds to STING dimers localized at the ER membrane, which leads to profound conformational changes that trigger STING oligomerization and liberation from anchoring factors. STING also can be activated by cGAMP from bacterial and GJIC sources, which establishes a role for STING as an independent pattern recognition receptor. Next, STING translocates from the ER to the Golgi apparatus through ERGIC. During transportation, STING recruits TBK1, thus promoting TBK1 autophosphorylation, and the phosphorylation of STING at the C-terminal tail, which provides a binding site for IRF3. The cGAS–STING signaling pathway not only induces type I IFN and inflammatory signaling responses but also activates other cellular processes including autophagy and apoptosis.

Pattern recognition receptors (PRRs) serve as innate immune sensors of danger signals, including pathogen-associated molecular patterns (PAMPs) and danger-associated molecular patterns (DAMPs), and activate the cellular stress response [1]. DNA, as PAMPs or DAMPs, can be sensed by PRRs to alert cells about the presence of microbial pathogens as well as of damaged or malignant cells and trigger innate immune responses [2]. The cGAS–STING pathway, comprising the cyclic GMP–AMP synthase (cGAS) and the stimulator of interferon genes (STING), was discovered as an important DNA-sensing machinery in innate immunity and pathogenic defense [3,4,5]. The cGAS–STING axis can be activated by both non-self DNA and self DNA including microbial DNA, released mitochondrial DNA, extra nuclear chromatin, cytosolic micronuclei, and aberrant chromosomal DNA (Figure 1). Upon binding DNA, cGAS produces cyclic GMP–AMP (2′3′-cGAMP) that binds to and activates the adaptor protein STING [6,7]. Activated STING translocates from the endoplasmic reticulum (ER) to the Golgi apparatus via the endoplasmic reticulum–Golgi intermediate compartment (ERGIC) [8]. In the meantime, activated STING proteins form large oligomers and recruit the downstream TANK-binding kinase 1 (TBK1) via the TBK1 binding motif (TBM) at the C-terminal tail (CTT). The CTT-bound TBK1 phosphorylates the serine in the STING motif pLxIS (Ser365 in mouse and Ser366 in humans) at the CTT domain, which provides a binding site for interferon regulatory factor 3 (IRF3) [9]. IRF3, upon phosphorylation by the nearby TBK1, forms a dimer and translocates to the nucleus, inducing type I IFNs (IFN-I) and other cytokines. After activation by STING, TBK1 and its homolog IκB kinase epsilon (IKKε) activate the transcription factor nuclear factor κB (NF-κB) through the IKK complex [10].

Recently, several studies reported that the intrinsic cGAS–STING pathway not only facilitates IFN-I and inflammatory production, but also activates other cellular processes such as autophagy [1,11]. The cGAS–STING pathway could induce autophagy in a variety of species, including mammals, Drosophila, and sea anemone (*Nematostella vectensis*). The cGAS–STING pathway was shown to be responsible for the activation of autophagy in human cells with extra chromosomes, such as in cells from individuals with Down syndrome or other aneuploidy-associated pathologies [12]. Studies in herpes simplex virus type1 (HSV-1)-infected murine embryonic fibroblasts showed that HSV-1 infection significantly increased LC3 punctum formation and autophagic flux through the cGAS–STING pathway [13]. A research in Zika virus-infected Drosophila (*Drosophila melanogaster*) brain indicated that Zika virus induced LC3 lipidation through the STING pathway, which induced antiviral autophagy to limit Zika virus in the Drosophila brain [14]. It also suggested that autophagy is an ancient and highly conserved function of the cGAS–STING pathway, which predates the emergence of the type-I interferon pathway in vertebrates [8]. STING from the sea anemone (*Nematostella vectensis*) induces autophagy but not interferon in response to stimulation by cGAMP, which suggests that autophagy is a primordial function of the cGAS–STING pathway [8].

Several studies have provided evidence showing that autophagy could be induced by cGAS directly without any involvement of STING during DNA virus infections [15,16]. However, other studies have revealed that autophagy could be activated by STING after stimulation by cGAMP or poly(dA:dT) [13,17]. Besides, many studies reported that the cGAS–STING pathway induces a classical and canonical pathway-associated autophagy, whereas other researches indicated that the cGAS–STING pathway induces a non-canonical pathway-associated autophagy [13,18]. Given the complex mechanisms used by the cGAS–STING pathway to activate autophagy, several crucial and meaningful questions naturally arise: How does the cGAS–STING pathway regulate autophagy? What kind of autophagy is induced by the cGAS–STING pathway? Why does the cGAS–STING pathway trigger autophagy? What are the effects of cGAS–STING-induced autophagy? Thus, the aim of this review is to summarize the available mechanisms and the consequences of cGAS–STING-activated autophagy.

## 2. Canonical and Non-Canonical Autophagy

Autophagy is an evolutionally conserved, highly regulated catabolic process that not only degrades and recycles long-lived proteins, but also eliminates dysfunctional or broken organelles including mitochondria, peroxisomes, and ribosomes [21]. Currently, three main types of autophagy are recognized, including macroautophagy, microautophagy and chaperone-mediated autophagy, according to the different ways by which the cellular content is incorporated into lysosomes. Among these, macroautophagy is the most prevalent and best studied type, and is usually referred to as canonical autophagy. It is characterized by the delivery of cytoplasmic cargo to the lysosomes, which depends on the hierarchically ordered activity of autophagy-related (Atg) proteins recruited at the phagophore assembly site to form an autophagosome and ultimately fuse with the lysosomal compartment [22]. In contrast to canonical autophagy, recent findings suggested that macroautophagy can also occur in the absence of some key autophagy proteins through the unconventional biogenesis of autophagosomes. This type has been defined as non-canonical autophagy [23].

**Figure 2:**
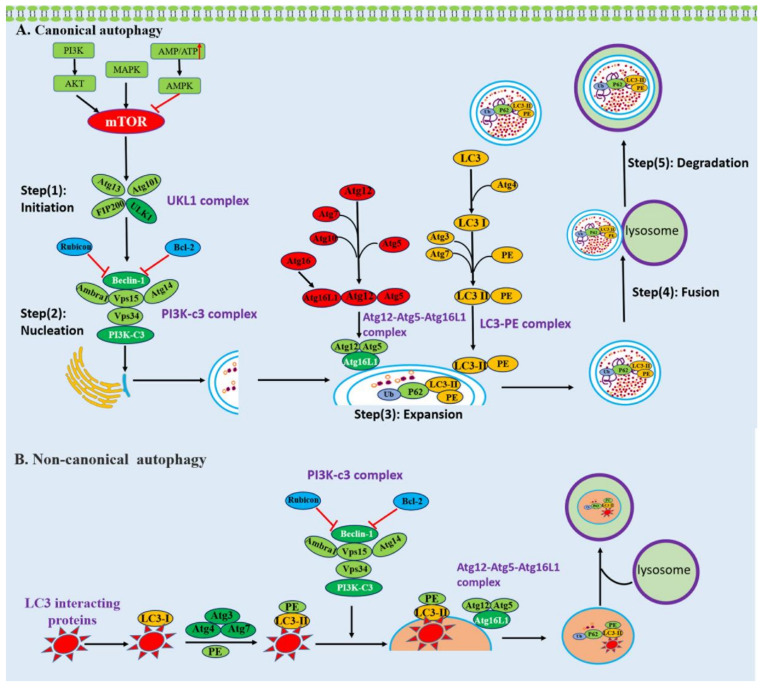
Schematic presentation of canonical and non-canonical autophagy. (**A**) The process of canonical autophagy. Canonical autophagy can be roughly divided into five steps: (1) initiation, (2) nucleation, (3) expansion, (4) fusion, (5) degradation and is mediated by Atg family proteins, kinases, and lipid metabolism. (**B**) The process of LAP-like non-canonical autophagy. LAP-like non-canonical autophagy is initiated by autophagy receptors through their LIR interacting with LC3. LC3 is recruited to single-membrane phagosomes. LAP-like non-canonical autophagy is independent of the upstream autophagy regulators mTOR and ULK1 initiation complex, while it needs the class III PI3KC3 complex and members of the protein lipid conjugation complex.

Canonical autophagy is initiated by various cellular stresses and can be roughly divided into five steps: (1) initiation, (2) nucleation, (3) expansion, (4) fusion, (5) degradation. It is controlled by Atg family proteins, kinases, and lipid metabolism (Figure 2). The initiation step is triggered by inhibiting mechanistic target of rapamycin kinase (mTOR) or activating AMP-activated protein kinase (AMPK), leading to the assembly and activation of the ULK1 complex including ULK1, Atg13, Atg101, and FIP200 [24,25,26]. The step of nucleation requires the ULK1 complex to phosphorylate the class III PI3KC3 complex which consists of the tumor suppressor Beclin-1, Beclin-1-regulated autophagy protein 1 (AMBRA1), the lipid kinase VPS34, the scaffolding protein VPS15, and the autophagy-specific subunit Atg14 [27]. The ATG12–ATG5–ATG16L1 complex and LC3–phosphatidylethanolamine (PE) are necessary for promoting the expansion of the phagophore membrane [28]. After autophagosome formation, autophagosomes undergo a stepwise maturation process including fusion events with multivesicular endosomes and lysosomes. Fusion with lysosomes is necessary for the complete degradation of the segregated cytoplasm [29].

Non-canonical autophagy is also a key cellular pathway in immunity, characterized by the conjugation of LC3 to endolysosomal membranes such as phagosomes and endosomes [30]. Non-canonical autophagy is associated with endocytic and engulfment processes, including LC3-associated endocytosis (LANDO) and LC3-associated phagocytosis (LAP), which have essential functions in the clearance of pathogens and cytokines and in responses to lysosome biogenesis. During the process of LANDO and LAP, LC3 coats the surface of endosomes and phagosomes, respectively, regulating the trafficking and lysosomal clearance of engulfed extracellular materials [31]. The canonical and non-canonical autophagy pathways share overlapping machineries, but there are essential differences (Figure 2). First of all, canonical autophagy is initiated by the formation of a double-membrane phagophore known as the isolation membrane, which engulfs the cytosolic cargo. Compared to canonical autophagy, non-canonical autophagy is characterized by a single-membrane phagosome [32,33]. Furthermore, the non-canonical pathway is independent of the upstream autophagy regulators mTOR and ULK1 initiation complex; nevertheless, it needs the class III PI3KC3 complex and members of the protein lipid conjugation complex [34]. Additionally, the WD (tryptophan–aspartic acid) repeat-containing C-terminal domain of ATG16L is essential for LC3 recruitment to endolysosomal membranes during non-canonical forms of autophagy but is dispensable for canonical autophagy [35].

## 3. Molecular Mechanisms of cGAS–STING Pathway-Induced Autophagy

### 3.1. cGAS Can Directly Interact with Beclin-1 to Induce Autophagy

Liang and collaborators indicated that cytoplasmic DNA could trigger autophagy and recruit LC3-positive autophagic vesicles by promoting cGAS to interaction with Beclin-1 [15]. cGAS contains an amino (N)-terminal regulatory domain (RD, a.a. 1–160), a central nucleotidyl transferase (NTase) domain (a.a. 161–330), and a carboxyl (C)-terminal domain (CTD, a.a. 331–522) [36]. The central region of Beclin-1 contains a Bcl-2 binding domain (BD, a.a. 112–159) for cellular and viral Bcl-2 interactions, a coiled-coil domain (CCD, a.a. 142–270) for Atg14L and UVRAG interaction, and an evolutionarily conserved domain (ECD, a.a. 245–450) for PI3KC3 (Vps34) interaction [15,37]. It was found that the central NTase domain of cGAS and the central CCD of Beclin-1 are responsible for their interactions [15].

**Figure 3:**
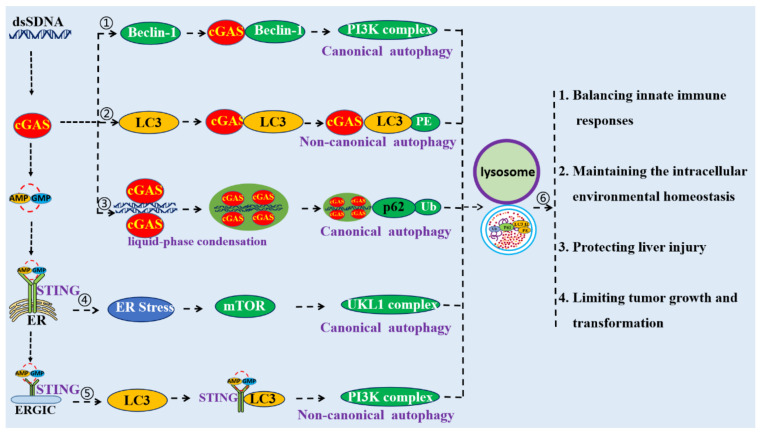
Schematic mechanism of cGAS–STING pathway-mediated autophagy. ① cGAS could directly interact with Beclin-1 to induce autophagy. ② cGAS could directly interact with LC3 to induce autophagy. ③ cGAS–dsDNA polymers can form a liquid-phase condensate, which could be an initiator of autophagy. ④ STING could induce autophagy through its interactions with LC3. ⑤ STING triggers autophagy through the ER stress–mTOR pathway. ⑥ Autophagy induced by the cGAS–STING pathway is crucial for the modulation of innate immune responses, to maintain intracellular environmental homeostasis, to protect against liver injuries, and to limit cellular transformation and tumor growth.

The interaction between cGAS and Beclin-1 increases the level of autophagy by competing with the binding of Rubicon to Beclin-1 (Figure 3). Beclin-1 is a core component of the Beclin-1–PI3KC3 complex, a lipid–kinase complex involved in autophagosome nucleation [38]. The activity of the Beclin-1–PI3KC3 complex is tightly regulated by many factors, especially different binding partners [39]. Atg14L and Rubicon associate with the Beclin-1–PI3KC3 complex; Atg14L positively regulates autophagy at various steps, whereas Rubicon negatively regulates both autophagy and endocytosis at the membrane fusion step by suppressing PI3KC3 lipid kinase activity [15,40]. It has been shown that Rubicon is stably associated with Beclin-1 complexes in 293T cells. Upon stimulation by dsDNA, Rubicon progressively dissociated from the Beclin-1–PI3KC3 complex, while the association between Atg14L and the Beclin-1–PI3KC3 complex was not affected [15]. Since both cGAS and Rubicon competitively bind to the first two CCDs of Beclin-1, increasing the cGAS–Beclin-1 interaction could cause the decrease of the Rubicon–Beclin-1 interaction [15]. Taken together, the interaction of cGAS with Beclin-1 leads to the dissociation of the negative autophagy factor Rubicon from the Beclin-1 complex, initiating the activation of PI3KC3 kinase and thereby increasing autophagy.

### 3.2. cGAS Can Directly Interact with LC3 to Induce Autophagy

The abundance of proteins and cellular organelles is usually controlled by selective autophagy, a process mediated by autophagy receptors that simultaneously bind the cargoes and LC3 proteins on phagophores’ membranes. Studies have shown that cGAS is a receptor that induces selective autophagy by interacting with LC3 [18]. LC3-interacting regions (LIRs) are short linear motifs within selective autophagy receptors, which can bind to LC3 and induce autophagy [41]. cGAS was found to contain a total of five LIRs, two of which are conserved in different species [18]. The LIR motif of cGAS mediates the interaction with LC3, and deletion of LIR355–360 or mutation of the evolutionarily conserved phenylalanine 357 and valine 360 impairs the interaction of cGAS with endogenous LC3 [18]. Additionally, it was indicated that Atg14 and Atg7 are involved in the process of autophagy induced by cGAS [18]. Atg14, a subunit of the PI3K complex, localizes to isolation phagophores and is essential for autophagosome formation during the process of canonical autophagy [42]. ATG-7 is an E1 ubiquitin-activating enzyme required for the activation of the ATG12–ATG5–ATG16L1 complex, which is crucial for phagophore elongation and autophagosome biogenesis during canonical autophagy. These studies have suggested that cGAS could trigger non-canonical autophagy by interacting with LC3, and this process shares machineries with canonical autophagy, such as the PI3K complex and members of the protein lipid conjugation complex (Figure 3).

### 3.3. The cGAS–dsDNA Polymer Can Form a Liquid-Phase Condensate, Which Could Be an Initiator of Autophagy

An accumulating amount of DNA in the cytoplasm could activate cGAS and drive the formation of liquid-like droplets, which allows cGAS to detect the presence of DNA in the cytoplasm above a certain threshold and trigger a switch-like response [43,44]. When cGAS is activated by DNA ligands, it assembles into a dimer, in which the DNA strands are sandwiched between two cGAS protomers [3]. Each cGAS protomer contains two principal DNA binding sites, named A and B. In the dimer, two dsDNA strands are bound with two cGAS, while one dsDNA binds to site A of one cGAS protomer, and the other dsDNA strand binds to site B of the other cGAS protomer [3]. The formation of the initial 2:2 cGAS–DNA complex prearranges the two DNA duplexes in a roughly parallel fashion, and a recent study has indicated that multiple cGAS dimers can form a ladder-like assembly with long DNA ligands [45]. DNA binding to cGAS induces a robust phase transition to liquid-like droplets, which function as microreactors in which the enzyme and reactants are concentrated to drastically enhance the production of 2′3′-cGAMP [43]. Several studies suggested that liquid-like droplets of cGAS could provide a mechanism that prevents cGAS activation by a low amount of genomic or mitochondrial DNA that might be present in the cytoplasm at different cell cycle stages [46]. Because cGAS cannot distinguish foreign DNA from self-DNA, it relies on liquid-phase separation to measure the concentrations of cGAS and DNA in the cytoplasm and mount a switch-like immune response only when the concentrations of these molecules rise above a certain threshold [46].

Increasing evidence suggests that the liquid-like condensate is a main regulator of both bulk and selective autophagy [47]. Liquid-like protein droplets undergo internal rearrangement and can recruit other molecules, with a potential to host biochemical reactions. Therefore, it is possible that thanks to its liquid-like properties, the condensates could provide a platform for recruiting autophagy-related molecules such as LC3, p62, and Atg proteins that drive autophagosome formation [48,49]. In this pathway, the liquid-like properties of condensates gradually form larger aggregates, are labelled by ubiquitin, and eventually become autophagosomes and are degraded when they fuse with lysosomes [48,50]. Further, it was suggested that liquid-like condensates play important roles in multiple steps of autophagy, mediating the assembly of autophagosome formation sites, acting as a modulators of TORC1-mediated autophagy regulation, and triaging protein cargos for degradation [47].Thus, autophagy could be initiated by the Liquid-Phase Condensate formed by The cGAS-dsDNA Polymer (Figure 3).

### 3.4. STING Can Induce Autophagy by Interacting with LC3

It was found that STING is an autophagy receptor that can directly interact with LC3 via its LIR motifs to mediate autophagy [13]. Co-IP experiments and pulldown analyses have shown that STING and LC3 could physically interact with each other [13]. The iLIR web server was used to analyze the STING protein sequence, and it was revealed that STING contains seven putative LIRs (a.a. 40–52, 76–88, 100–112, 161–173, 180–192, 193–205, and 239–251) [13]. It has been demonstrated that three LIRs (a.a. 161–173, 193–205, and 239–251) are involved in the interactions between STING and LC3 [13]. Further studies have indicated that the process of STING-induced autophagy requires the activation of STING by phosphorylation [8]. Activated STING could leave the ER, moving to the ERGIC and Golgi in a process dependent on the COP-II complex and ARF GTPases [51]. Vesicles budding from the ER and ERGIC could serve as membrane sources for LC3 lipidation and autophagosome biogenesis [52]. Thus, it is possible that autophagy induced by STING requires the membrane fraction derived from ER or ERGIC as the substrate for LC3 lipidation. It was shown that treatment with Brefeldin A (BFA), a specific inhibitor of protein trafficking, specifically inhibited protein trafficking from ER to ERGIC by targeting ARF GTPases. Under this condition, STING-stimulated cells lost the ability to stimulate LC3 lipidation [8]. Therefore, the process of budding from ER is required for STING-induced autophagy.

It was reported that autophagy induced by STING is independent of upstream autophagy regulators such as Beclin-1, ULK1, and Atg9a, but dependent on downstream autophagy regulators such as Atg5 and Atg16L1 [13,17]. It was also shown that knockout of Beclin-1, ULK1, or Atg9a failed to block poly(dA:dT) and STING-induced LC3-II conversion and punctum formation. However, knockdown of Atg5 could block STING-induced LC3 punctum formation and autophagic flux in HeLa cells [13]. Additionally, STING activation induced LC3 lipidation onto single-membrane perinuclear vesicles, mediated by ATG16L1 via its WD40 domain, bypassing the requirement of the canonical upstream autophagy machinery [17]. These studies suggest that STING initiates non-canonical autophagy by interacting with LC3 (Figure 3).

### 3.5. STING Triggers Autophagy through the ER Stress–mTOR Pathway

Wu and collaborators indicated that STING was involved in the activation of ER stress and unfolded protein response (UPR) [53]. The accumulation of unfolded or misfolded proteins in the ER could result in ER stress, which triggers the UPR to alleviate it and restore ER homeostasis [54]. The process of UPR is relayed through three ER transmembrane sensors, i.e., IRE1α, PERK, and ATF6, and these UPR sensors are typically bound to the key ER chaperone GRP78 to prevent downstream signaling [55]. When unfolded protein stress reaches a critical threshold, GRP78 can dissociate from these sensors thus allowing downstream signaling activation that regulates transcription and translation and restores ER homeostasis [56]. It was shown that co-transfecting cGAS and STING into HEK293T cells to transiently activate STING signaling could upregulate UPR gene expression including the levels of Activating transcription factor 3 (ATF3) and Growth arrest and DNA damage-inducible protein 34 (GADD34) mRNAs, which are markers of ER stress and UPR [53]. It has been indicated that live bacteria could elicit an ER stress response through c-di-AMP to activate the STING pathway [57]. Further, STING resides in the ER where it maintains ER calcium homeostasis through its interaction with the Ca^2+^ sensor stromal interaction molecule 1 (STIM1) [58]. Additionally, it was shown that STING is a crucial regulator of ER stress, as the protein expression levels of p-PERK, p-IRE-1α, and p-eIF2α were markedly stimulated by aortic banding surgery; however, these results were markedly restrained in STING-KO mice [59].

It has been observed that STING mediated ER stress and the UPR through a novel motif, named “the UPR motif” [53]. Structural and functional analyses demonstrated that the UPR and IFN are mediated through distinct domains of STING. The helix (a.a 322–343) of STING, specifically, residues R331 and R334, is critically required for STING-mediated UPR. Deletion of the helix from full-length STING (Δ322–343) could abrogate STING-mediated GADD34 induction [53].

STING triggers autophagy through the ER stress–mTOR pathway. Autophagy is negatively controlled by the mTOR signaling pathway [60]. mTOR is a downstream effector of the PI3K/AKT pathway and forms two distinct multiprotein complexes, namely, mTORC1 and mTORC2 [61]. mTORC1 is a key inhibitor of autophagy activated by diverse stimuli such as growth factors, nutrients, energy, and stress signals to control cell growth, proliferation, and survival; however, the function of mTORC2 in autophagy is controversial [62]. It was shown that ER stress induces autophagy through a negative regulation of the AKT–mTOR pathway [63]. The PERK–CHOP pathway activated by ER stress could mediate the induction of TRB3, which can directly bind to AKT and suppress the AKT–mTOR signaling pathway. Additionally, one study indicated that bacterial c-di-AMP activated STING and triggered ER stress, leading to mTOR inactivation and subsequent autophagy in macrophages [57]. These studies suggest that STING could trigger autophagy through the ER Stress–mTOR pathway (Figure 3).

### 3.6. Role of TBK1 and IRF3 in cGAS–STING Pathway-Induced Autophagy

Accumulating evidence indicates that TBK1 and IRF3 are dispensable for STING-induced formation of the autophagosome [9]. The CTT of STING (a.a 341–379) is required and essential for recruiting TBK1 and IRF3. It was shown that deletion of the CTT from STING (Δ341–379) could impair the downstream phosphorylation of TBK1 and IRF3 but did not affect LC3 lipidation and the interaction between STING and LC3 [9]. The S365A mutation of STING is defective in phosphorylation, thereby disrupting the recruitment of IRF3 while retaining the ability to recruit TBK1. A STING mouse strain with a mutation at position 365 (S365A) showed a disruption in IRF3 binding and the resulting absence of type I interferon induction but not of autophagy [9]. The mutation L373A of STING prevents the recruitment of TBK1 and the activation of both IRF3 and NF-κB [64]. STING knock-in mice carrying the mutation L373A of STING present normal LC3 lipidation. Additionally, RNA interference (RNAi) to knock down the STING downstream kinase TBK1 and the transcription factor IRF3 had no obvious effects on STING-induced autophagy. Neither TBK1 nor IRF3 deficiency blocked the increase of LC3-ΙΙ levels and LC3 punctum formation induced by poly(dA:dT) stimulation [64].

TBK1 is not involved in the formation of autophagosomes but is implicated in their maturation and degradation. Prabakaran and collaborators indicated that STING was not degraded by autophagy in TBK1-deficient cells [65]. TBK-1 knockdown did not affect the formation of autophagosomes but suppressed their maturation [65]. It was noticed that TBK-1 is a key regulator of immunological autophagy and is responsible for the maturation of autophagosomes [65]. TBK1 mediates maturation and degradation of autophagosomes through the following mechanisms. Firstly, it was reported that TBK-1 could phosphorylate the autophagic adaptor p62 on Serine 403, which increased the affinity of p62 for ubiquitin chains [66]. After STING stimulation, p62 and phospho-TBK1 showed significant co-localization, and phospho-TBK1 was recruited to p62 in stimulated cells [65]. Secondly, it has been shown that in addition to p62, TBK1 could bind to another autophagy receptor, i.e., NDP52 to form the NDP52–ULK–TBK1 complex, which is essential for the growth of WIPI2-positive phagophores and LC3-positive autophagosomes to drive xenophagy [67]. Thirdly, TBK1 could regulate microtubule dynamics in mitosis and the cytoplasmic levels of dynein. The maturation of autophagosomes transport to lysosome-rich areas occurs through microtubules and is dependent on the action of the motor protein dynein [68]. It has been shown that the loss of TBK1 could impair microtubule transport and then the maturation of autophagosomes into autophagolysosomes [69].

## 4. Functions of Autophagy Induced by the cGAS–STING Pathway

Autophagy induced by the cGAS–STING pathway is crucial for preventing excessive immune response and persistent immune stimulation. Autophagy induced by cGAS–STING could modulate the innate immune responses through the degradation of cGAS and STING and the elimination of DNA and pathogens from the cytosol. cGAS-induced autophagy through its interaction with LC3 and Beclin-1 not only suppresses the synthesis of 2′3′-cGAMP to halt type I IFN production, but also enhances autophagy-mediated degradation of cGAS and DNA to prevent excessive cGAS activation and persistent immune stimulation [15,70]. STING can be degraded by autophagy through TBK1-mediated p62 phosphorylation to drive STING ubiquitination and autophagic degradation that could attenuate the innate immune signaling [65,70]. Autophagy is thus able to control infections, as it functions as an intracellular defense mechanism capturing cytosol-invading pathogens and driving them to lysosomal degradation [71]. During *Mycobacterium tuberculosis* or HSV-1 infections, cytosolic pathogenic DNA triggers cGAS–STING pathway-mediated autophagy to eliminate the pathogens [72].

Further, autophagy induced by the cGAS–STING pathway is crucial for maintaining intracellular environmental homeostasis [73]. The cGAS–STING pathway induced autophagy through the ER stress pathway, which could in turn alleviate ER stress and restore homeostasis via isolating and sequestering it [74]. Moretti and colleagues revealed that after sensing c-di-AMP derived from Gram-positive bacteria, STING triggered ER stress, mTOR inactivation, and ER-phagy and eventually curtailed cell death [57]. It was also suggested that autophagy induced by the cGAS–STING pathway plays a crucial role in micronuclei homeostasis [18,75]. cGAS reduced the abundance of micronuclei through the induction of autophagy, cleaning micronuclei and preventing chromosomal instability [18]. cGAS is a crucial regulator of selective autophagy to clear the micronuclei caused by genotoxic stress [18]. Knockdown of cGAS along with induction of mitotic arrest in HeLa and U2OS cancer cells clearly resulted in an increase of micronuclei formation and chromosome mis-segregation [75]. Amar and his collaborators suggested that STING-mediated autophagy is protective against H_2_O_2_-induced cell death [73]. STING-mediated autophagy serves as a protective mechanism to remove damaged cells and promotes a protective environment in MEFSV40 cells after treatment with H_2_O_2_ [73].

Additionally, it was found that autophagy induced by the cGAS–STING pathway exerts a protective role in liver cancer, limiting transformation and tumor growth [16]. cGAS-mediated autophagy protects the liver from ischemia/reperfusion (I/R) injury, which is independent of STING. Deletion of cGAS suppressed hypoxia-induced autophagy in hepatocytes and thus led to apoptotic cell death during liver I/R [16]. Autophagy is a therapeutic strategy in cancer, through the modulation of both therapeutic resistance and the death of cancer cells [76]. STING activation induced by host DNA damages can trigger autophagy-dependent cell death to remove cancer cells or other stressed cells [25]. Guendalina and colleagues suggested that oncolytic viruses can successfully activate antitumor immunity via the activation of a STING-dependent antiviral cascade in cancer cells [77].The nucleoside analog zalcitabine (an antiviral drug) induces mitochondrial damage and the release of mtDNA into the cytosol, resulting in the activation of the cGAS–STING pathway, which in turn induces autophagy-dependent ferroptosis and suppresses pancreatic tumor growth in mice [78]. The release of DNA from the nucleus to the cytosol caused by nuclear cathepsin B (CTSB)-mediated genomic DNA damage activates STING-dependent autophagy and leads to ferroptotic cell death in human pancreatic cancer cells [79].

## 5. Conclusions and Future Perspective

The cGAS–STING pathway triggers autophagy through both canonical and non-canonical pathways. It triggers canonical autophagy through the interactions of cGAS and Beclin-1, the liquid-phase separation of the cGAS–DNA complex, and STING-triggered ER stress–mTOR signaling. It also triggers non-canonical autophagy through the binding of cGAS and/or STING to LC3. Autophagy induced by the cGAS–STING pathway is crucial for balancing innate immune responses, maintaining intracellular environmental homeostasis, preventing liver injury, and limiting tumor growth and transformation.

It was reported that the process of budding from ER is required for STING-induced autophagy; however, whether STING transportation from ERGIC to Golgi is involved in the process of STING-induced autophagy is unknown. Further, it was suggested that IRF3 is not involved in the formation of autophagosome induced by the cGAS–STING pathway but is essential for DNA-stimulated degradation of STING by autophagy [65]. However, the molecular mechanisms activated by IRF3 in STING degradation are not resolved. Thus, it is recommended that studies should be designed to explore these aspects. Additionally, evidence has shown that the cGAS–STING pathway not only activates autophagy but also induces apoptosis [80]. Apoptosis can play a key role in the innate response to pathogenic infection [81]. It is recommended that the relationships between apoptosis, autophagy, and IFN responses induced by the cGAS–STING pathway draw more attention.

## Data Availability

Not applicable.
